# Methodological development of a pneumatic artificial muscle-driven left ventricular simulator using interpretive structural modeling

**DOI:** 10.1371/journal.pone.0355859

**Published:** 2026-08-13

**Authors:** Turgut Batuhan Baturalp, Selim Bozkurt

**Affiliations:** 1 Department of Mechanical Engineering, Whitacre College of Engineering, Texas Tech University, Lubbock, Texas, United States of America; 2 School of Engineering, Ulster University, Belfast, United Kingdom; University of Verona: Universita degli Studi di Verona, ITALY

## Abstract

Mock circulation systems can replicate a wide range of cardiac operating scenarios, such as cardiovascular diseases, particularly muscular dysfunctions. These systems are operated by sophisticated control algorithms which require complex driver and data acquisition systems, whereas animal models raise ethical concerns. Therefore, there is a need for novel testbeds to overcome the challenges in the existing systems. The aim of this study is to develop a less complex and realistic left ventricular (LV) simulator. The Interpretive Structural Modeling (ISM) approach was utilized to evaluate different left ventricular actuation methods and materials. ISM revealed that the most suitable method to actuate the LV is using the pneumatic artificial muscles and latex rubber. The performance of the new LV simulator was evaluated for healthy and diseased conditions at 50, 60, 70 and 80 bpm heart rates. The experiments revealed that the beating LV simulator can generate average flow rates of up to 2.25 L/min. The actual human LV-like wall motion was also verified by the twisting angle on the apex of the beating LV simulator with 21 degrees of rotation and 11 mm of apex shortening. The prototyped beating LV simulator is a promising preliminary platform and may reach actual human LV’s hemodynamic and cardio-mechanical performance with further improvements.

## Introduction

Mock circulatory systems are hydraulic devices consisting of electromechanical components and simulate blood pressures and flow rates in the cardiovascular system [1]. They have been widely used in developing and testing cardiovascular implants, such as artificial heart valves and heart pumps, before marketing [2] as the international standard requires testing of the hydrodynamic performance of such implants [3]. Moreover, anatomical models can be installed on these devices to simulate patient-specific conditions [4]. Physiological cardiovascular signals are generated in mock circulatory systems in chambers representing ventricles and pressurized by pistons, which are controlled to follow a reference position as in the commercial Vivitro pulse duplicators [5] or pressure trajectories as described by Schampaert et al. [6]. Time-varying elastance functions or single-fiber contraction models have been used to simulate reference trajectories in the mock circulatory systems being controlled to follow pressure signals [7,8]. The ventricular chambers in these systems can be silicone sacks [5] or can be made of rigid materials [8]. Nonetheless, ventricular volumes are simulated by the stroke of the pistons over each cardiac cycle and measured by tracking the piston motion [5,8]. The aortic compliance and resistance are modeled by air chambers and throttle valves to simulate the aortic pressure and flow rates [9]. Although mock circulatory systems also allow the installation of ex-vivo components, such as cardiac valves and the evaluation of complex scenarios, such as papillary muscle repair [10], adding such components to these systems increases the complexity of the design and operating conditions [11].

Hybrid mock circulatory systems consist of an interface including a numerical model that simulates hemodynamic variables such as flow rate or volume and a physical section in which pressures are simulated [9]. An example of a hybrid mock circulatory system is presented by Ochsner et al. [12] and used to test ventricular assist devices. However, the design and development of hybrid mock circulatory systems present challenges such as fast and accurate numerical-physical interfaces or hardware drivers required to operate these devices [9].

Ex-vivo heart platforms offer realistic anatomical models to evaluate clinical scenarios and cardiac implants [13,14]. Active beating heart platforms include an explanted heart from animals, such as a porcine heart [15]. Explanted hearts are oxygenated, and electrical activity is restored; however, the heart rate is not regulated in these hearts due to a lack of the autonomic nervous system, and the natural rhythm of the heart might be remarkably different from physiological conditions [16]. Passive beating heart platforms again include explanted porcine hearts however, pressure and flow rate signals in these cardiac models are generated using pistons as in the mock circulatory systems [17]. Nonetheless, increasing ethical concerns about the use of animal models in research and established roadmaps to reduce animal experiments [18] may negatively affect the use of ex-vivo cardiac models.

More recent studies suggest the use of flexible material and anatomical models to simulate left ventricular function. For instance, Rocchi et al. [19] tested a platform simulating patient-specific anatomy made of 3D-printed phantoms with a combined in silico lumped-parameter model to reproduce pressure–volume relations. A limitation of the patient-specific framework is its workflow complexity, as clinical image segmentation, geometric reconstruction, and customized fabrication may increase time, cost, and technical requirements and may limit scalability. Davies et al. [20,21] report a bioinspired soft robotic left ventricle simulator that uses thin-filament artificial muscles to mimic multilayered myocardial biomechanics, reproducing physiological pressure and volume profiles in both healthy and failure conditions within a mock circulation loop. Soft-robotic systems with fully synthetic valve simulators rely on artificial actuators and complex actuation mechanisms with data-driven controllers to reproduce the cardiac contraction [21]. Although this system provides a soft robotic model capable of simulating complex cardiac motion and valve interactions, the fabrication and experimental setup remain highly specialized and complex, potentially limiting rapid reproducibility and wider adoption in routine benchtop studies or clinical research environments.

Beating heart simulators activated with artificial muscles offer possibilities to overcome the challenges in the aforementioned cardiac models [22]. Moreover, anatomical models can be molded and produced, whereas the use of artificial muscles can replicate cardiac contraction and relaxation over a cardiac cycle. However, designing a beating left ventricular (LV) simulator that replicates the geometry, wall motions, and muscle fiber orientations of a real human LV chamber is a complex problem [23–25]. This is because abnormal left ventricular wall motion is associated with health problems such as arterial hypertension, diabetes mellitus, coronary artery disease, and cardiac arrhythmia in elderly individuals [23], and may increase the risk of stroke recurrence [26]. Moreover, the shape of a healthy left ventricle resembles an ellipsoid, whereas it becomes more spherical in systolic heart failure, and myocardial fiber orientation is impaired [24]. Evaluation of clinical scenarios and different therapies for physiological conditions require realistic LV simulators. Therefore, design and operating factors in such simulators must be selected carefully to obtain realistic results. The novelty of the developed LV simulator is as follows. Methodologically, this study introduces Interpretive Structural Modeling (ISM) as a systematic framework for evaluating alternative ventricular actuation concepts and identifying the hierarchical relationships among design parameters prior to prototype development. Conceptually, the simulator shifts from the conventional piston-driven reproduction of ventricular hemodynamics toward a bio-inspired replication of myocardial mechanics, including ventricular torsion and longitudinal shortening. Technologically, the proposed platform integrates helically oriented pneumatic artificial muscles embedded within a deformable latex ventricular wall, enabling physiologically relevant twisting and contraction without the need for complex electromechanical drive systems, active feedback controllers, or highly specialized fabrication processes. Compared with existing mock circulatory systems and recent soft-robotic ventricular simulators, the proposed design aims to offer a relatively simple LV simulator with lower complexity.

## Materials and methods

In this study, the ISM methodology proposed by Warfield [27,28] is used to deal with complex design issues of the beating LV simulator. ISM provides a fundamental understanding of how various design parameters (elements, variables, system components, etc.) relevant to a design problem are interrelated and thus helps researchers to structure them in a meaningful manner, develop collective intelligence, and overcome complex problems. The goal of ISM is to develop a relationship map that includes paths of ideas and threads of thought to transform unclear and poorly expressed models into a visible, well-defined, and relatively easily solvable model [29]. The ISM method was applied to three different beating LV simulator design approaches (pneumatic artificial muscle, flexible band, and artificial muscle actuation) to compare and analyze the interaction of factors and their effects on the performance of the LV simulator. The first step of the ISM procedure is to utilize the knowledge of experts in the field to build a model that represents a complex system. A literature survey was conducted to construct the knowledge of experts to build the ISM model for different design approaches for the beating LV simulators. The ISM framework used in this study was constructed through a structured synthesis of both literature-based knowledge and expert judgment. First, a comprehensive literature survey was conducted focusing on mock circulation loops, left ventricular simulators, cardiac biomechanics, and actuation technologies for biomimetic cardiac devices. Key design factors influencing ventricular simulator performance—such as geometric fidelity, wall motion characteristics, controllability, actuation efficiency, structural integrity, and integration with the circulatory loop—were identified from prior studies and compiled into a list of candidate system elements. These factors were then evaluated using expert knowledge derived from researchers with experience in transdisciplinary system design, peer-reviewed publications, and interpretive structural modeling methodologies. Pairwise relationships between the identified factors were assessed to construct the Structural Self-Interaction Matrix, following the standard ISM procedure. The relationships captured in the Structural Self-Interaction Matrix were then converted into reachability matrices, transitivity was incorporated, and hierarchical levels were determined to generate the final ISM digraph. This process enabled the systematic representation of interdependencies among design parameters and facilitated the evaluation of alternative actuation approaches for the left ventricular simulator.

The main factors affecting design were categorized into two different groups with respect to their specifications for each LV simulator design approach: (a) common factors that exist in all design approaches, and (b) independent factors. Common factors are validly existing factors in all design approaches since they are related to the operating nature of human LV such as contraction rate and force of muscle fibers, controllability and preload ability of LV chamber, and common muscle fiber installation issues, like ease and adjustability of muscle fiber placement in the tissue material and structural integrity of muscle fibers with the tissue material of the LV chamber. Ease and adjustability of installation were included due to the shared influence for versatility and applicability of the actuation device. On the other hand, independent factors include the factors that vary and are distinctive for each LV simulator design approach.

### Design factors used to develop the beating LV simulator

In this section, common and independent design factors used to develop the beating LV Simulator are reviewed. Common factors include the contraction rate of myocardial fibers, the contraction force of myocardial fibers, the controllability of LV motion, the preload ability of LV, and the ease and adjustability of installation and structural integrity of the actuators with other components. Independent design factors include pneumatic artificial muscle actuation, flexible bands actuation, and artificial muscle actuation of the beating LV simulator.

#### Common design factors in the LV simulator.

The contraction rate of the cardiomyocyte fibers influences various parameters on the contraction or pumping performance of the LV chamber, such as LV twist and untwisting rate, heartbeat per minute, etc. [30]. The connection between afterload and muscle fiber shortening rate is an inverse relationship. Additionally, the effect of the inotropic state of the cardiac fiber on the force-velocity relationship was mentioned as a shift up and right in the force-velocity curve, such that there is an increase in both maximum velocity and isometric force with increasing inotropy. That means boosting force generation capability by the actin and myosin filaments and improving the rate of generation with an increasing rate of cross-bridge turnover [31]. Also, a relation was stated between the LV untwisting rate and the peak twisting angle as well as LV end-systolic volume and ejection fraction [32]. Geyer et al. [33] describe the shear strain as the amount of distortion associated with the sliding of plane layers over each other. Evaluation of deformation on LV can be described in two ways: (1) a motion around a given point in tissue in terms of space and time and (2) the interaction between the strain rate and contraction performance.

The myocardial deformation is described as follows: The ventricular muscle fibers contract in the longitudinal and circumferential dimensions, increase the wall thickness in the radial dimension, and twist along the long axis of the LV during cardiac systole [34]. The torsional behavior of LV is related to muscle contraction. whereas torsional rotation was highly related to transmural gradients of contractility [35].

The magnitude and timing of the LV torsion change the performance of the LV. The timing and function of the LV torsion can be defined as a measurement derived from the twisting or wringing motion around the long axis of LV [36]. The torsional movement stores energy for releasing at diastole to help ventricular filling which requires good timing of the cardiac muscle contractions. Another aspect of the controllability of LV motion or muscle contractions is the ability to mimic cardiomuscular dysfunctions [37]. Also, the time difference between peak rotations of the inner and outer layers of the LV wall was found to be significant and the pressure rate measurements matched with LV wall torsion [38].

The preload ability of the LV chamber can be explained by the Frank-Starling Law which expresses that the heart can increase its contraction capability by boosting venous return in stroke volume. The cardiac myocytes and other cardiac tissues stretch with the pressure generated by the atrium right before the contraction of the heart. Thus, it can be measured by the length of muscle fibers at the end of the diastole phase. The material property of the cardiac tissues has an important role in the preload ability of the chamber. The relationship between LV material properties and its performance was investigated and found to be highly related [39,40]. The main factors that affect LV preload can be listed as [31]: (1) LV compliance impacts the preload ability of the LV directly because higher compliance causes higher ventricular filling and more stretched myocytes; (2) Heart rate affects the LV preload by changing the filling time. Therefore, a higher heart rate adversely affects ventricular preload; (3) Most of the LV filling occurs with minimal contribution of atria at resting heart rates. Therefore, the assistance of atrial contraction can be neglected if the heart rate is in the normal resting heart rate range. Atrial contraction plays an important role when the heart rate escalates to the exercise range. The duration of the LV filling reduces significantly for high heart rates; (4) A weakening in the contraction of LV may create an increase in the preload ability due to the inability of blood ejection in normal volumes, which causes blood accumulation in LV and an increase in the preload ability of LV.

The installation of muscle fibers or actuators must be easy to adjust because the orientation and location of the muscle fibers on the LV wall play an important role in the contraction or pumping performance of the LV. Additionally, the actuators/muscle fibers should be able to contract with the connecting tissue in both the systole and diastole. The helical ventricular myocardial band concept was developed to define principal, cumulative vectors, by taking into account the tissue architecture/structure and net forces developed within the ventricular mass [41,42].

#### Independent design factors in the LV simulator.

Pneumatic Artificial Muscles are pressurized flexible air chambers that can contract by expanding in diameter with a pressurized air supply. The main advantages of Pneumatic Artificial Muscle are great force/weight ratio, low cost, flexible structure, and lightweight. Circumferential and longitudinal contraction and torsional deformations of the LV wall were investigated using a prototype made of plastic [43]. The performance of the prototyped LV chamber was found that the wall motion resembles a real LV and gave comparable results to human hemodynamic parameters. Pneumatic Artificial Muscles were also used on a mock LV chamber pump [44]. The developed Pneumatic Artificial Muscles were embedded in a silicon matrix tissue and attempted to mimic the motion of the LV by varying the applied pressure and configuration of Pneumatic Artificial Muscles.

Strings or flexible bands can also be used as muscle-like mechanisms for beating LV simulators by pulling them with the help of linear or servo motors. Hanson et al. and Alazmani [45,46] developed hybrid heart simulator systems to simulate the mechanical properties of the beating LV. They suggested that the heart simulator should be able to simulate normal and pathological heart motion at rates of up to 150 bpm, pressure should be sensed and should be able to represent the motion of the heart slice by slice. The device was actuated by electromagnetic swing-arm actuators which are controlled by a computer. The proposed flexible bands or strings require to be guided since they are pulled by an external force. The guidance creates a need for structural components that might not follow complex LV wall motions.

Electroactive polymers and biometal muscles are strong actuator candidates for the artificial muscle actuation approach. Electroactive polymers are of low cost and have suitable elastic properties and, in addition, can be manufactured in various sizes and shapes [47]. Other actuator candidates are materials such as nitinol and aramidic fibers that are flexible and capable of creating smooth movements. Biometal fibers on a carbon fiber scaffold can be used to create bi-ventricular assist devices [48]. Although these devices are not sufficient to replace the pumping function, they are used as assistive devices. Heat generation of biometal fibers is the primary design problem which requires extra components to dissipate the generated heat. Additionally, fatigue of the artificial muscles may affect the rate and force of contraction since they need to be stimulated at least 50 times per minute.

The common and independent factors affecting the beating LV simulator design approaches are compiled and given in Table 1.

**Table 1:** The main factors affecting the beating LV Simulator design approaches.

		Pneumatic Muscle Actuation	Flexible Bands Actuation	Artificial Muscle Actuation
**COMMON FACTORS**	1	Contraction Rate of Fibers	Contraction Rate of Fibers	Contraction Rate of Fibers
	2	Contraction Force of Fibers	Contraction Force of Fibers	Contraction Force of Fibers
	3	Controllability of LV Motion	Controllability of LV Motion	Controllability of LV Motion
	4	Preload Ability of LV	Preload Ability of LV	Preload Ability of LV
	5	Ease and Adjustability of Installation	Ease and Adjustability of Installation	Ease and Adjustability of Installation
	6	Structural Integrity with Other Tissues	Structural Integrity with Other Tissues	Structural Integrity with Other Tissues
**INDEPENDENT FACTORS**	7	Expansion in Perpendicular Direction	Grade of Guidance of Flexible Muscles	Heat Generation
	8	Limited Elastic Property	Fatigue Bands	Fatigue of Muscle Fibers
	9	Bending Force Generation Fibers	Bending Force Generation Fibers	Response Time of Muscle Fibers

**Fig 1:**
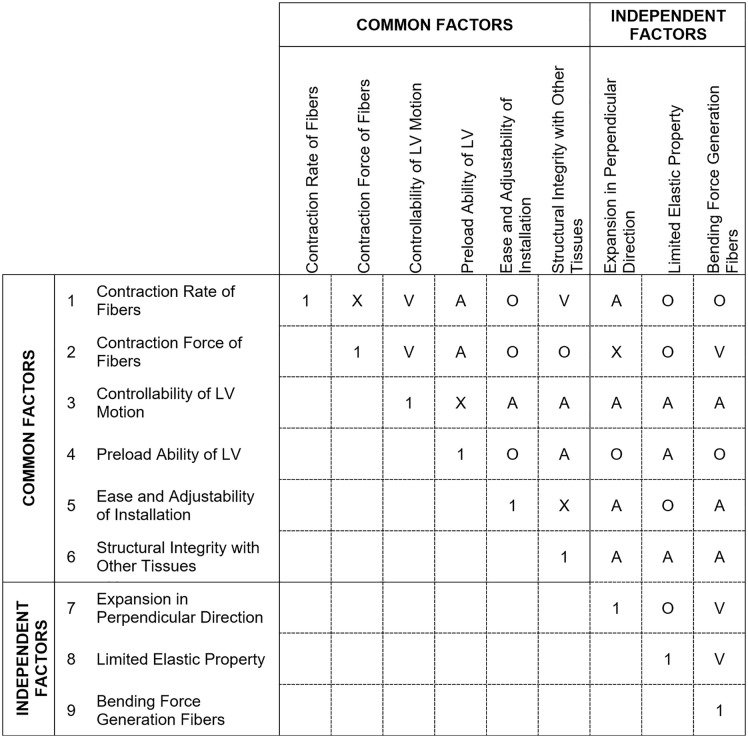
Structural Self-Interaction Matrix of pneumatic artificial muscle actuation approach, the functions of these symbols are described as: “V” stands for events when the row element influences the column element; “A” stands for events when the column element influences the row element; “X” stands for events when the row and column elements influence each other; “O” stands for events when there is no relationship between the row and column elements.

The Structural Self-Interaction Matrix was also developed by establishing contextual relationships for the pneumatic artificial muscle actuation approach after identifying the factors affecting LV design as given in Fig 1.

**Fig 2:**
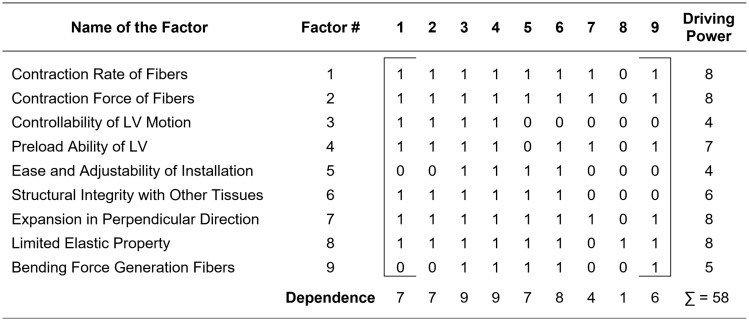
Reachability Matrix with the transitivity of pneumatic artificial muscle actuation approach.

The weak relationship between factors is neglected in the Structural Self-Interaction Matrix. This assumption is crucial for simplifying the complex system in decomposition. Letter coding is used in the Structural Self-Interaction Matrix to provide an easier input user interface and verbally define the interactions between factors. Also, the Structural Self-Interaction Matrix is converted into a binary matrix which includes only 1 and 0 as elements to facilitate the required matrix calculations by ISM analysis. These binary numbers answer the question of “yes” or “no”. After the interaction matrix is converted into a binary matrix, the transitivity rule is included in the analysis to establish transitivity between factors. The transitivity rule states that, if factor A is related to factor B and if factor B is related to factor C, then factor A is related to factor C. Therefore, not only direct but also indirect interactions between factors can be represented in a reachability matrix that embraces transitivity. The reachability matrix with the transitivity of the pneumatic artificial muscle approach is given in Fig 2. Driving power and dependence of factors are also computed as shown in Fig 2. The summation of column values gives the dependence of the corresponding factor, while the summation of the row values gives the driving power.

While the calculated driving force and dependence of each factor give an idea about the classification of factors into groups, the final reachability matrix with antecedents of each factor is used to reveal the level partition [28]. The group positions are determined by the separation of the antecedent set and the reachability set. From these two sets, the intersection set is determined. The factors common in the reachability set and the antecedent set are included in the intersection set. These three sets help to identify the level of the factors. If all the factors of the intersection and reachability sets of any particular factor are the same, then that factor is identified as the top-level group (level I) in the ISM hierarchy. Once the top-level factors are identified, it is removed from the set to identify the next level. The factors, along with their reachability set, antecedent set, intersection set, and levels, are shown in Table 2.

**Table 2:** Level partition of factors in pneumatic artificial muscle actuation approach.

Factor Number	Reachability Set	Antecedent Set	Intersection Set	Level
1	1 2 3 4 5 6 7 9	1 2 3 4 6 7 8	1 2 3 4 6 7	
2	1 2 3 4 5 6 7 9	1 2 3 4 6 7 8	1 2 3 4 6 7	
3	1 2 3 4	1 2 3 4 5 6 7 8 9	1 2 3 4	**I**
4	1 2 3 4 6 7 9	1 2 3 4 5 6 7 8 9	1 2 3 4 6 7 9	**I**
5	3 4 5 6	1 2 5 6 7 8 9	5 6	
6	1 2 3 4 5 6	1 2 4 5 6 7 8 9	1 2 4 5 6	
7	1 2 3 4 5 6 7 9	1 2 4 7	1 2 4 7	
8	1 2 3 4 5 6 8 9	8	8	
9	3 4 5 6 9	1 2 4 7 8 9	4 9	
1	1 2 5 6 7 9	1 2 6 7 8	1 2 6 7	
2	1 2 5 6 7 9	1 2 6 7 8	1 2 6 7	
5	5 6	1 2 5 6 7 8 9	5 6	**II**
6	1 2 5 6	1 2 5 6 7 8 9	1 2 5 6	**II**
7	1 2 5 6 7 9	1 2 7	1 2 7	
8	1 2 5 6 8 9	8	8	
9	5 6 9	1 2 7 8 9	9	
1	1 2 7 9	1 2 7 8	1 2 7	
2	1 2 7 9	1 2 7 8	1 2 7	
7	1 2 7 9	1 2 7	1 2 7	
8	1 2 8 9	8	8	
9	9	1 2 7 8 9	9	**III**
1	1 2 7	1 2 7 8	1 2 7	**IV**
2	1 2 7	1 2 7 8	1 2 7	**IV**
7	1 2 7	1 2 7	1 2 7	**IV**
8	1 2 8	8	8	
8	8	8	8	**V**

**Fig 3:**
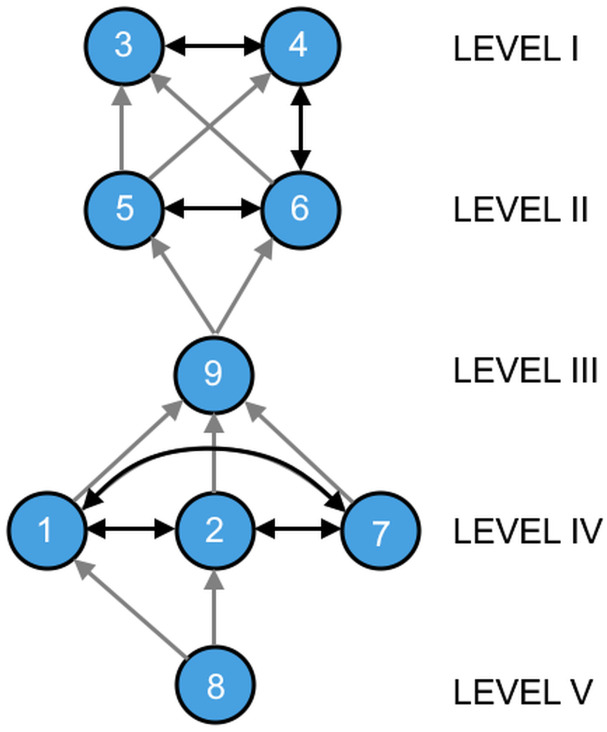
Relationship network digraph of the pneumatic artificial muscle actuation approach.

The levels given in Table 2 help to build the digraph formation. The formation of a digraph reveals the association of sets and binary relations through matrices by converting them into a graphical form [49]. Relationship arrows are extracted from the reachability matrix with transitivity by inspecting the selected factor’s relation with the factors around it. The directions of the arrows can be found by examining the reachability matrix with transitivity row by row (from the selected factor to the surrounding factors), and column by column (from the surrounding factors to the selected factor). Finally, the digraph is converted to ISM to see a broad representation of the interrelationship between the factors (Fig 3).

**Fig 4:**
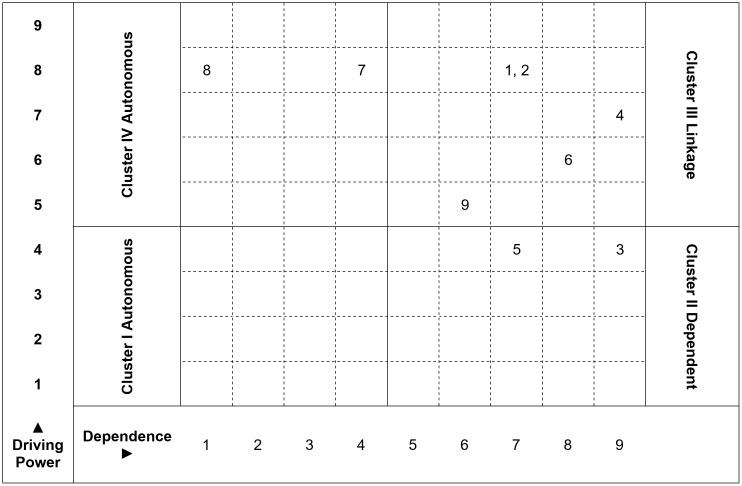
MICMAC analysis of the pneumatic artificial muscle actuation approach.

The final step for the ISM model is developing the MICMAC analysis. MICMAC (Matrice d’impacts croisés multiplication appliquée á un classement) Analysis is a cross-impact matrix multiplication for classification method used in structural prospective analysis (ISM) to examine indirect relationships The MICMAC analysis was developed to study the diffusion of impact through reaction paths and loops for developing hierarchies for members of an element set [50]. The purpose of the MICMAC analysis is to arrange the factors with respect to their driving power and dependence in four clusters. These four clusters are defined as autonomous, dependent, linkage, and independent factors [51]. The driving power and dependence of the factors are obtained from the final reachability matrix, and the factors are positioned in a MICMAC chart as given in Fig 4.

**Fig 5:**
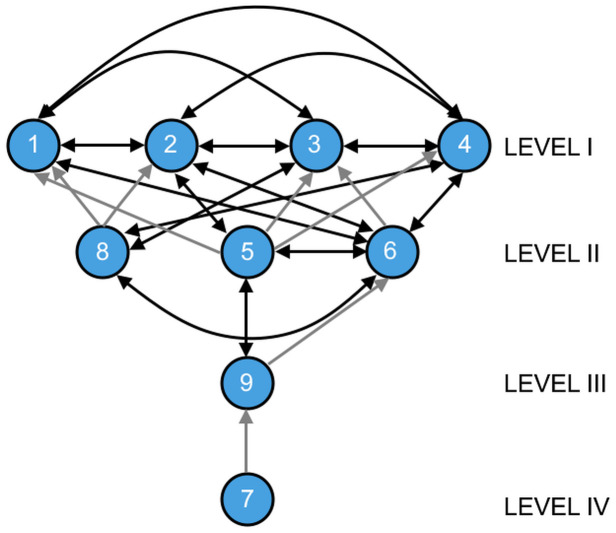
Relationship network digraph of the flexible band actuation approach.

**Fig 6:**
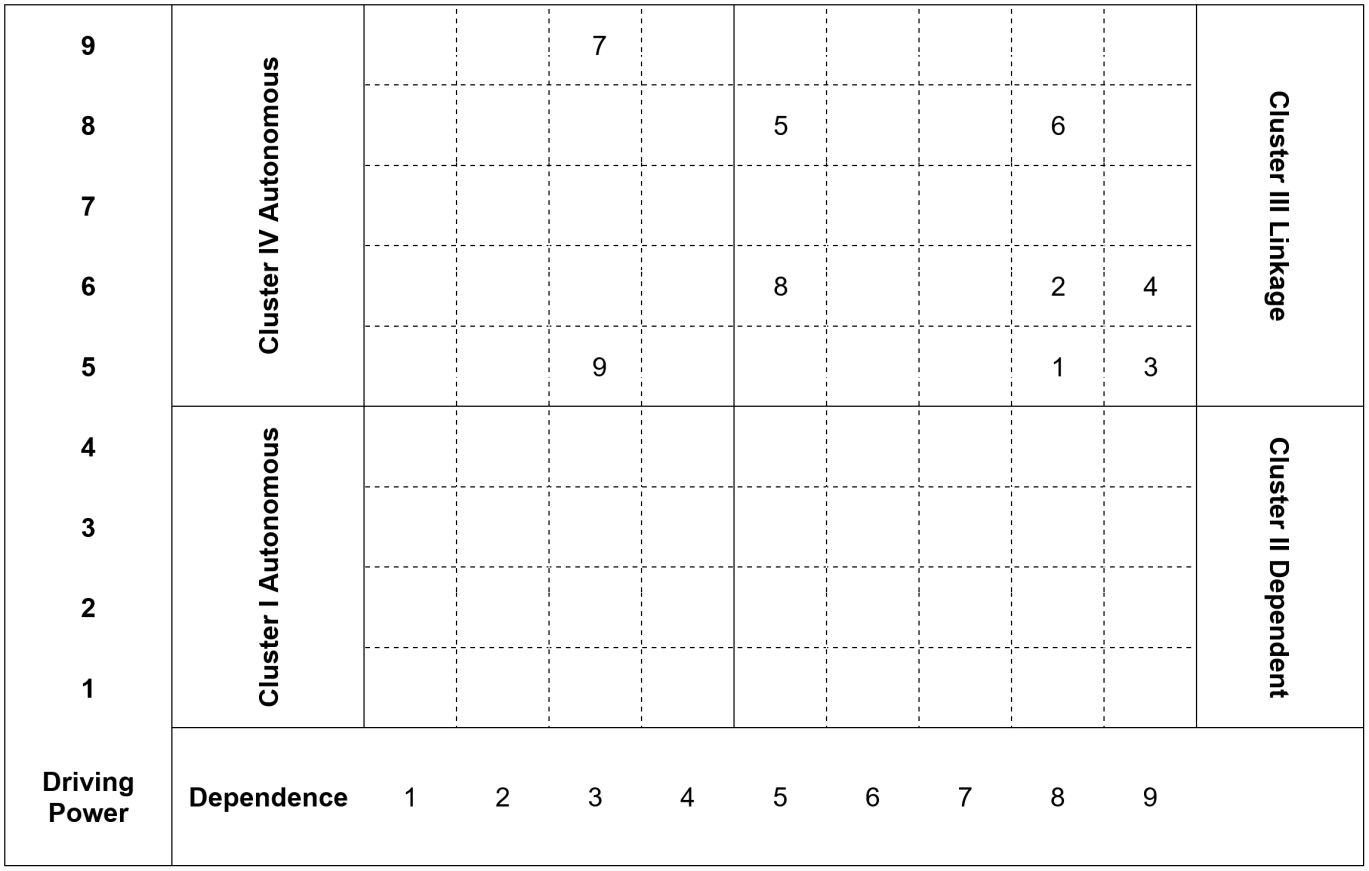
MICMAC analysis of the flexible band actuation approach.

**Fig 7:**
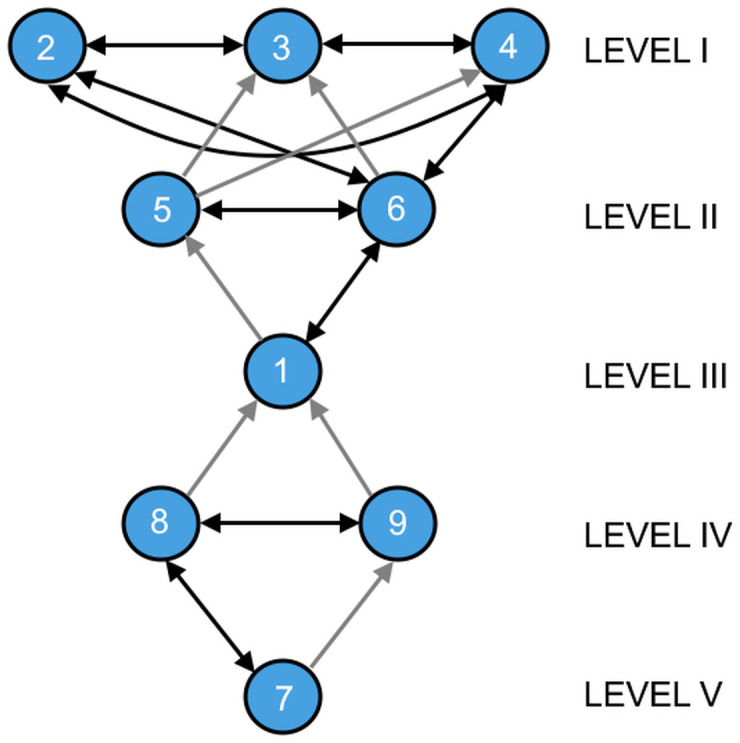
Relationship network digraph of the artificial muscle actuation approach.

**Fig 8:**
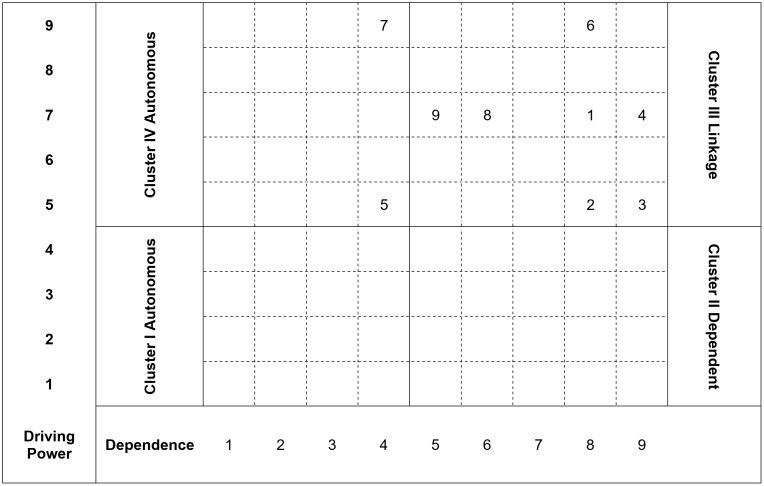
MICMAC analysis of the artificial muscle actuation approach.

The same ISM method was used to develop the relationship network digraph and the MICMAC analysis for the flexible bands and artificial muscle actuation design approaches (Figs 5–8).

All performance measures of factors affecting the performance of the beating LV simulator have been classified into four categories in MICMAC analyses. Cluster I includes autonomous factors. They have low driving power and low dependence; hence, they can be eliminated or ignored in the design process. Since this is a complex design problem with a high interaction level, none of the factors is classified as autonomous, which indicates that there is no disconnected factor for all three design approaches. Cluster II includes dependent factors that have low driving power and high dependence. Only the pneumatic artificial muscle approach has factors in this region, which are controllability of LV motion (Factor 3), and ease and adjustability of installation (Factor 5). While factors 3 and 5 have a smaller guidance power compared to other factors, the controllability of LV motion is extremely dependent on other factors of the pneumatic artificial muscle approach. Cluster III includes linkage factors where most of the factors are placed for all three designs. This indicates that most of the design factors for all approaches have high driving power and high dependence. In terms of design terminology, any action on the factors in the linkage cluster in the design process may result in affecting the entire performance of the design. Therefore, designing such systems is a complex task. The number of factors can be considered as an indication of the complexity of the system. While the pneumatic artificial muscle approach has five factors in this region, flexible band, and artificial muscle actuation approaches have seven factors. Therefore, it can be concluded that the pneumatic artificial muscle approach is less complex than the other approaches. The major factors within Cluster III that influence the device performance must be carefully analyzed and integrated with the whole projected system. Cluster IV includes independent factors with a strong drive power but very weak dependence.

The independent factors with high driving power are identified as factors 8 and 7 for pneumatic and artificial muscle actuation designs, respectively. As a reminder, factor 8 is called limited elastic property, and factor 9 is called heat generation. The limited elastic property factor can be defined as the overall elasticity limitation of the beating LV simulator by introducing actuation components. Although introducing any additional materials, including artificial muscles, will reduce the elasticity of the beating LV simulator, the stiffness of the pneumatic muscles is considerably higher than artificial muscles. Yet, it can be compensated by choosing the right orientation of actuation components. The artificial muscle components are actuated using electricity, which generates significant heat. This generated heat should be dissipated for the next cycle of actuation for stable performance. Unfortunately, cycle frequencies are notably short for the beating LV simulator application, for example, the generated heat should be dissipated in less than 0.5 seconds to have a 60 bpm normal heart rate.

Another comparison was made based on relation network digraphs. Fig 5 shows that there are multiple interactions between many different factors (1, 2, 3, 4, 8, 5, 6) which makes the flexible bands actuation design approach more complex than the other two approaches. A similar comparison can be made between Figs 3 and 7, which reveals that the pneumatic artificial muscle design approach is a less complex system design than the artificial muscle actuation design approach. Therefore, the pneumatic artificial muscle approach was chosen to go further in the development of the beating LV simulator.

### Prototype production of the LV simulator

The LV simulator was built using natural liquid mold-making latex rubber from AeroMarine Products Inc. The tensile stress of the latex rubber used in the LV model is around 21 MPa whereas its maximal strain is around 863 mm/mm [22]. The LV geometry was designed considering anatomical ranges of basal diameter and long-axis length in a healthy heart. Also, a conic geometry was used to take out the mold from the LV model after curing. The range of the LV basal diameter is between 39 mm and 56 mm and the long axis length is between 90 mm and 104 mm [52–54]. Basal diameter was selected as 45 mm and long axis length was selected as 97 mm. The LV diameter was reduced along the long axis to 40 mm resulting in 118 mL left ventricular pressure-free volume size. The 3D-printed inner mold was brushed with liquid latex rubber to create a thin layer of the inner LV chamber surface.

The pneumatic artificial muscles consist of two main materials: the flexible inner tube and the braided sleeve. Super-soft latex rubber tubing was used for the inner tube due to its very flexible material properties (Durometer 40A). While the inner diameter of the selected tube is 1/16 in, the outer diameter is 1/8 in. The inner tube was covered with a braided mesh sleeve to control its lateral expansion and to direct the motion to the axial contraction. An expandable polyester sleeving was used with an inner diameter of 1/4 in, whereas the expanded inner diameter was 7/16 in. One end of the pneumatic artificial muscles was sealed, and the other end was connected to the air supply.

**Fig 9:**
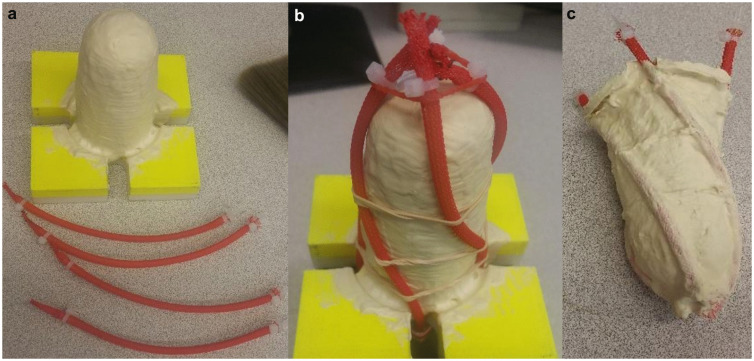
(a) Four pneumatic artificial muscles and first layer application of the latex rubber on the 3D printed mold, (b) positioning of the pneumatic artificial muscles on the latex rubber, (c) completed beating left ventricle chamber.

Fig 9a shows the applied thin layer of latex rubber on the 3D printed mold and four constructed pneumatic artificial muscles. After a thin layer of latex rubber was applied to the inner mold, the pneumatic artificial muscles were placed in helical orientation (Fig 9b) to increase the pumping performance with the help of torsional contraction and mimic the swirling pattern of the cardiac muscles. Four artificial muscles were used from base to apex, covering a 90-degree angle on the LV geometry, so as not to make the LV chamber too stiff while ejecting liquid at around 2 L/min. The integration between the pneumatic artificial muscles and tissue material was achieved by adding latex rubber layers. Specific design decisions, including the number of actuators, their orientation, their spatial arrangement, and the selection of materials, are based on Finite Element Analyses. Detailed information about the specific design decisions can be found in [55]. The final product of the beating LV simulator prototype is shown in Fig 9c.

**Fig 10:**
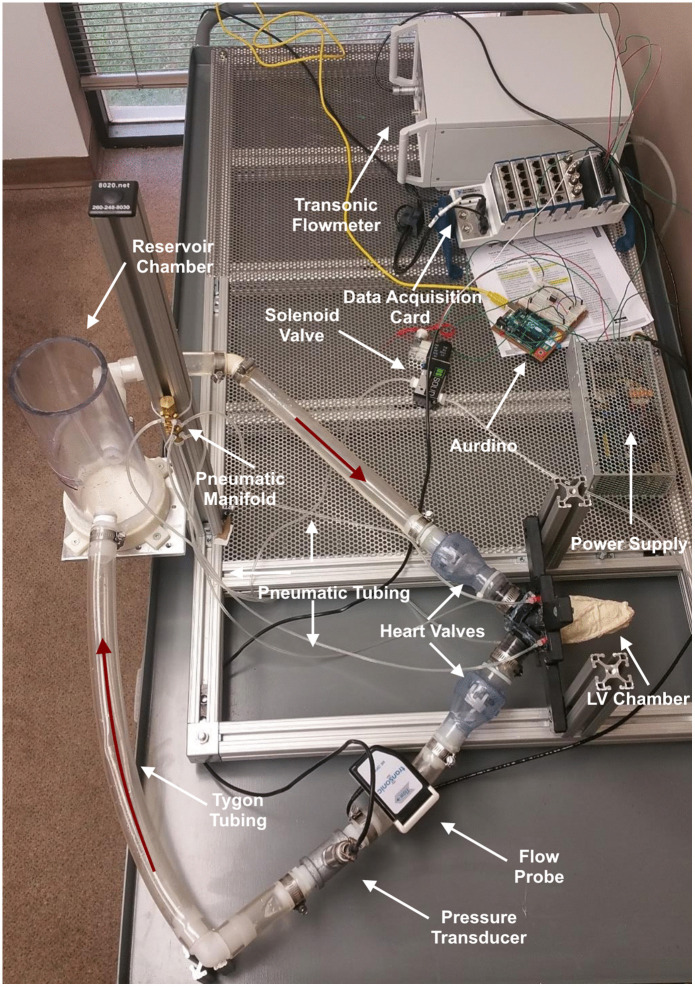
Mock circulatory setup for testing the performance of the developed beating left ventricular simulator.

A circulatory system consisting of swing flap type check valves as mitral and aortic valves, Tygon-3603 tubing with 1 in inner diameter, and an open-air reservoir as the left atrium, was built for preliminary testing purposes of the prototyped beating LV simulator. Pressure and ultrasonic flow rate sensors were used in the circulatory system, both connected right after the aortic valve. The pressure sensor was the PX-309 series from Omega Inc. and the flow rate measurements were taken by PXL-20 ultrasonic flow sensor from Transonic Inc. Both sensors were factory calibrated. The Transonic flow sensors can be used as as plug and play device on Transonic flowmeters. For the pressure sensor, the calibration curve for the PX-309 series from Omega Engineering is used to convert the measured electrical output into the corresponding pressure value after the installation. The mitral and aortic valves were connected right after the inlet and outlet outputs of the beating LV simulator holder. The left atrium tank was mounted on an adjustable aluminum profile so that the atrium pressure could be varied in the experiments. A data acquisition device (DAQ) (NI-cDAQ-9178 with a NI 9215 module from National Instruments Labs Inc) was used to collect the analog voltages from the pressure and flow sensors. A three-way two-position type pneumatic electric solenoid was used to direct air supply to the beating LV simulator. The activation signal of the solenoid was provided with an Arduino Uno R3 microcontroller board so that the desired heartbeat action could be performed. An algorithm was written and embedded into Arduino for commanding the activation (open position) and deactivation (closed position) time intervals of the solenoid in terms of milliseconds. The solenoid was supplied with pressurized air of 80 psi to avoid damage in the LV chamber. A pneumatic manifold distributed the air to the pneumatic tubing to pressurize the pneumatic artificial muscles. A glycerin-water mixture with 40% glycerin and 60% water was used as the circulation fluid in the preliminary performance testing of the beating LV simulator. It is a suitable blood analogy fluid due to its density and viscosity [56,57]. The experiments were conducted for 50, 60, and 70 bpm heart rates. The cases of the experimental study were selected to examine not only the effect of heart rate but also the effect of systolic and diastolic phase durations and the effect of cardiac muscle dysfunction. Durations of systole and diastole were determined in two different ways: using the physiological ratio values [58] and dividing the whole heartbeat duration into two equal systolic and diastolic durations. Furthermore, the effect of cardiac muscle dysfunction was simulated by turning off one of the pneumatic artificial muscles on the beating LV simulator. Each setting was tested by performing a single run in the LV simulator. The flow rates and pressure values were measured at the aortic valve outlet. Mean aortic pressure and flow rate were calculated by averaging the pressure and flow over each cardiac cycle. More information about the circulatory system can be found in [59]. The configuration and components of the circulatory setup, and the flow direction, are shown in Fig 10.

## Results

**Fig 11:**
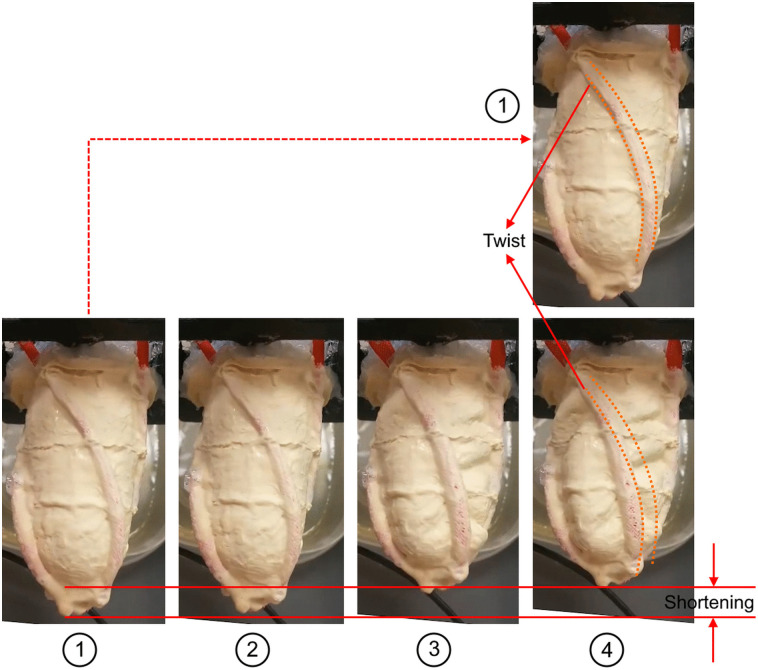
Representation of the twisting angle and longitudinal shortening of the beating left ventricular simulator (60 bpm heart rate and 0.5 s/0.5 s systolic-diastolic ratio).

Systolic and diastolic duration ratios were varied for each case, and a weakened LV case was simulated for 60 bpm. For the healthy 60 bpm with the physiological systole/diastole duration ratio, the contracted inner volume, which corresponds to end-systolic volume, was measured as approximately 85 mL. Therefore, stroke volume, the difference between relaxed and contracted volumes, was measured as 33 mL. The ejection fraction was calculated as 28%, which is lower than a healthy heart’s ejection fraction but highly promising for an initial beating LV simulator prototype. In addition to the ejection fraction, the twist angle and displacement of the apex were also measured. The longitudinal shortening of the ventricle was measured as 11 mm, and the twist angle at the apex was approximately 21 degrees. The twist angle and longitudinal shortening are shown in Fig 11, where four frames show twists over a cardiac cycle from the beginning of diastole to the end of systole in equal intervals, and the means of measured hemodynamic variables for each cardiac cycle are listed in Table 3.

**Table 3:** Mean flow rate and mean aortic pressure in the beating LV simulator and circulatory system for each cardiac cycle (S and D represent systole and diastole, SD and CV represent standard deviation and coefficient of variation).

Heart Rate and S/D Ratio	Mean Flow ± SD [L/min]	CV [%]	Mean Aortic Pressure ± SD [mmHg]	CV [%]
50 bpm (0.42 s/0.78 s)	2.03 ± 0.02	0.87	12.94 ± 0.06	0.46
50 bpm (0.60 s/0.60 s)	1.78 ± 0.03	1.69	12.86 ± 0.01	0.08
60 bpm (0.35 s/0.65 s)	2.01 ± 0.03	1.49	13.10 ± 0.03	0.23
60 bpm SICK (0.35 s/0.65 s)	1.47 ± 0. 02	1.36	12.92 ± 0.01	0.08
60 bpm (0.50 s/0.50 s)	2.08 ± 0.03	1.44	13.27 ± 0.02	0.15
70 bpm (0.315 s/0.540 s)	2.25 ± 0.03	1.34	12.96 ± 0.03	0.23
70 bpm (0.428 s/0.428 s)	2.01 ± 0.03	1.49	13.15 ± 0.14	1.06
80 bpm (0.375 s/0.375 s)	2.18 ± 0.03	1.38	13.14 ± 0.02	0.15
80 bpm (0.300 s/0.450 s)	1.71 ± 0.03	1.75	13.09 ± 0.02	0.15

**Fig 12:**
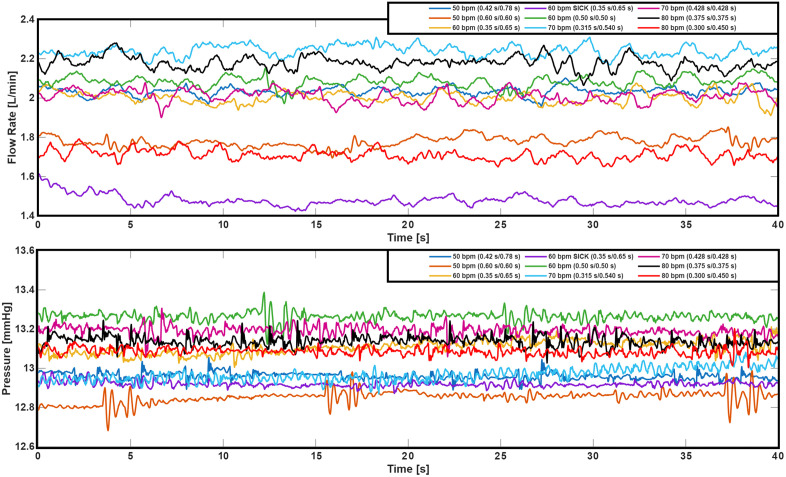
The moving average flow rates and pressures over time in the beating LV simulator and circulatory system.

The moving average flow rates and pressures over the duration of a cardiac cycle for different heart rates and systolic/diastolic ratios in the beating LV simulator and circulatory system are given in Fig 12.

**Fig 13:**
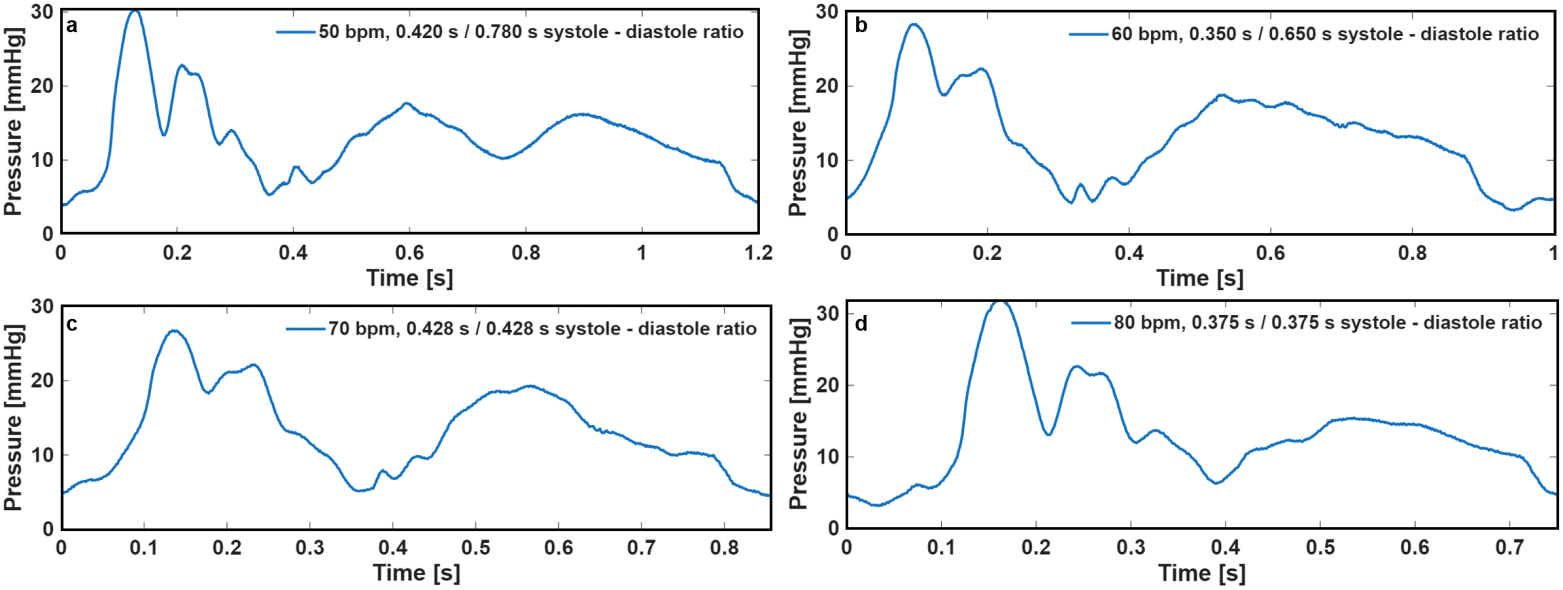
Aortic pressure waveforms in the LV simulator for (a) 50 bpm heart rate and 0.420 s/ 0.780 s systole ratio, (b) 60 bpm heart rate and 0.350 s/ 0.650 s systole ratio, (c) 70 bpm heart rate and 0.428 s/ 0.428 s systole ratio and (d) 80 bpm heart rate and 0.4375 s/ 0.375 s systole ratio.

The average flow rate increased with increasing heart rate. The simulated cases, such as the cardiac muscle dysfunction case (SICK), 70 bpm case with half/half systole/diastole durations, and 60 bpm case with physiological systole/diastole durations, reveal the importance of the systole/diastole duration ratio, which should be synchronized with the movement of the flow, or there must be sufficient time to fill the LV chamber. Aortic pressure waveforms in the LV simulator for 50 bpm heart rate and 0.420 s/ 0.780 s systole ratio, 60 bpm heart rate and 0.350 s/ 0.650 s systole ratio, 70 bpm heart rate and 0.428 s/ 0.428 s systole ratio and 80 bpm heart rate and 0.4375 s/ 0.375 s systole ratio are given in Fig 13.

Systolic aortic pressure was around 30 mmHg in the simulated heart rates, whereas diastolic pressure remained below 10 mmHg. Although the aortic pressure remained below physiological levels throughout the cardiac cycle, the pressure waveforms resembled physiological waveforms.

## Discussion

In this study, a new LV simulator was developed, and the hydrodynamic performance of the developed LV simulator was tested. The novelty of the developed system lies in the use of the ISM method, which was used to systematically evaluate alternative actuation mechanisms and analyze the interdependencies among design parameters. Conceptually, the developed LV simulator adopts a bio-inspired approach to ventricular mechanics and aims to reproduce the anatomical torsional twisting and radial contraction of the human left ventricle. Technologically, the use of the pneumatic artificial muscle–driven LV prototype was integrated into a circulatory loop. The use of helically oriented pneumatic artificial muscles enables the simulator to generate both ventricular contraction and rotational motion.

In this study, the design parameters of three different types of LV simulators were evaluated using the ISM approach. The ISM approach utilizes related elements in a structured way to deal with complex systems [60]. The motion of the actual LV is very complex and depends on various factors at the cellular, molecular, and organ levels, such as electrical activity, ion dynamics, blood pressure, wall thickness, etc. [61]. Therefore, a complexity decomposition tool such as ISM analysis is essential for the design process of beating the LV simulator. Alternative design methodologies, such as Analytical Hierarchy Process, Design of Experiments or Genetic Algorithms, were not selected to evaluate the design parameters. Because the Analytical Hierarchy Process does not explicitly model interdependencies among design factors [62]. However, the developed LV simulator design involves interactions between variables, and ISM explicitly models these interrelationships. Design of Experiments requires physical prototypes or simulations and does not handle conceptual design relationships [63]. Genetic Algorithms require mathematical objective functions and do not explain relationships between system variables [64]. ISM allows analysis of conceptual design options before physical experiments, making it more suitable for early-stage design evaluation.

The ISM method revealed that beating LV simulators actuated with pneumatic artificial muscles is less complex than beating LV simulators actuated with flexible bands or electroactive polymers. Therefore, the new beating LV simulator was built using pneumatic artificial muscles. The well-known design factors to achieve realistic LV performance, such as shape, wall motion, and actuation orientation, were not included in the ISM analysis. Because the transformation of the heart shape during the beating is directly related to the pumping performance of the LV [65]. The motion of the LV wall is, therefore, essential for the replication of the pumping function of the LV. Also, the cause of the torsional movement of the LV is possible due to the helical orientation of myocardial fibers [66–69]. The results showed that the beating LV simulator can mimic the LV wall movements and hemodynamic values of an actual human LV chamber. The experiments revealed that the beating LV simulator can generate average flow rates of up to 2.25 L/min with an average pressure of 15 mm Hg. The actual human LV-like wall motion was also verified by the twisting angle on the apex of the beating LV simulator with 21 degrees of rotation and 11 mm of apex shortening. The apical to the basal rotation angle of a healthy ventricle changes within a wide range; however, typical average values reported in the literature are around 16 deg to 18 deg [70–73] and can increase up to 24 deg [74]. The long-axis shortening in a healthy ventricle is around 10 mm [52] again changes within a range and can go up to around 14 mm [75]. Therefore, the LV simulator mimics the LV rotation and long-axis contraction close to a healthy ventricle.

The use of pneumatic artificial muscles eliminates the need for complex mechanical drive mechanisms, such as electric motors, gear assemblies, or bulky blocks connecting the components. Also, the control architecture is simpler compared to conventional electromechanical systems, as pneumatic actuation allows for smooth and compliant motion with fewer components. This simplified configuration offers advantages such as reducing mechanical complexity and minimizing potential points of failure. The LV simulator works with only four pneumatic muscles, reducing the complexity of the system. The reduced number of components lowers manufacturing and maintenance costs. Also, the system is lighter and more compact, making it easier to integrate into laboratory environments.

The personalized cardiovascular simulator presented in [19] simulates atria and the right ventricle using in-silico models, whereas the left ventricle is activated by electric motors and gear pumps. Therefore, this setup is operated somewhat similarly to pulse duplicators and can generate physiological cardiac outputs around 5 L/min and pressures around 110 mmHg. The soft robotic left heart simulator presented in [20,21] can generate systolic blood pressures above 100 mmHg; however, the duration of the diastolic phase remains shorter compared to the native left ventricle. The setup presented in [20,21] has 3 degrees of freedom. In the present study, each pneumatic muscle embedded within the left ventricle sack operates with one degree of freedom, again making it simplified and less complex. When pressurized air enters a pneumatic artificial muscle, it inflates. Due to the structural constraints of the surrounding latex rubber and the specific orientation of the muscle, the only input pressure in a channel causes contraction along a predefined path. This contraction results in multi-axial motion. When the air pressure is released, the elastic potential energy stored in the latex rubber forces the channel to deflate and return to its original resting state along that same path. The movement of each individual muscle is entirely dictated by a single variable, which is the internal air pressure, and is physically constrained to move along one specific trajectory. Therefore, it is kinematically classified as a one degree of freedom actuator.

The LV simulator developed in this study can generate flow rates around 2.3 L/min and 30 mmHg systolic blood pressure. However, it operates in a much simpler way and generates aortic pressure signal waveforms resembling physiological aortic pressure, as highlighted before. Cardiovascular devices such as implantable cardiac assist devices actuated with pneumatic artificial muscles have been proposed. For instance, Roche et al. [76] designed a soft robotic sleeve actuated by pneumatic artificial muscles, achieving circumferential and helical contraction. Although the actuation principle is similar in the beating left ventricular platform developed in this study and the soft robotic sleeve, the purpose of the devices is different. Therefore, there are differences in the structures of both devices, such as the left ventricular platform including and integrated mitral and aortic valves on it. Lorenzon et al. [77] proposed a left ventricle pump driven by inverse pneumatic artificial muscles. In this design, the inverse pneumatic artificial muscles drove the left ventricle with external helical contraction and achieved 70 mL stroke volume over 2 s, resulting in around 2.1 L/min left ventricular output. In the proposed design, the pneumatic artificial muscles were embedded in the left ventricular wall to contract the left ventricle model rather than squeezing it. Physiological heart rates between 50 bpm and 70 bpm were simulated, achieving a maximal cardiac output of around 2.25 L/min. While soft-robotic systems or 3D-printed phantoms require complex data-driven controllers, clinical image segmentation, geometric reconstruction, and highly specialized fabrication. The presented setup can be fabricated using latex rubber and 3D printed molds as shown in the figures making it a simpler and more easily reproducible platform for benchtop.

The reported pressure values are substantially lower than physiological human blood pressure, where arterial pressure changes between 80 mmHg and 120 mmHg [78]. The pressure values were low because of a lack of aortic compliance and resistance components in the circulatory loop. Lack of resistance in the system shifted the pressure values to the lower ranges, while lack of compliance components and the existence of the air bubbles in the system caused backflow and beating phenomena. In the human cardiovascular system, arterial compliance allows vessels to store and release energy during the cardiac cycle, while systemic vascular resistance contributes to the generation of physiological pressures [78]. As a result, the measured pressures reflect the mechanical pumping capability of the tested device within a low-load hydraulic environment rather than physiological conditions experienced by the native left ventricle. Therefore, the current results are useful for comparative evaluation and proof-of-concept testing within the developed LV simulator. However, they may not directly translate to physiological performance.

Ventricular pressures and volumes over a cardiac cycle in mock circulatory systems or pulse duplicators driven by pistons are simulated by the movement of pistons for healthy and pathological conditions. Therefore, the effects of the cardiac implants such as left ventricular assist devices, on the ventricular geometry or intraventricular septum cannot be simulated in these systems [79]. However, the developed beating LV simulator can simulate the interaction between the LV wall and the left ventricular assist device because the LV is made of a flexible material.

Piston motion in mock circulatory systems is achieved by sophisticated control algorithms [8,9] which require complex driver and data acquisition systems. The developed beating LV simulator operates in a relatively simple way. Air pressure is regulated in the solenoid valve controlled by the Arduino. Arduino controls the solenoid valve to achieve the desired systolic and diastolic ratios and heart. However, there is no active feedback control, as in a pulse duplicator, to achieve the piston trajectory. Contraction of the LV is achieved by the air pressure in the pneumatic artificial muscle and pathological conditions such as heart failure were simulated by reducing the number of active muscles in the simulator. This decreases the overall contractile force of the ventricular wall. As a result, the model exhibits reduced contraction efficiency, lower pressure generation, and altered ventricular dynamics that qualitatively resemble heart failure–like behavior. Disabling one or more pneumatic artificial muscles in the LV simulator represents the loss of contraction in specific regions of the ventricular wall. Pneumatic artificial muscles mimic myocardial fiber action, therefore, disabling an actuator simulates regional myocardial dysfunction. For example, cancelling one pneumatic artificial muscle corresponds to myocardial infarction or ischemia [80]. Disabling multiple pneumatic artificial muscles can represent heart failure with reduced ejection fraction, in which overall ventricular contractility is weakened [81]. It should be noted here that clinical validation is outside the scope of this study. Again, the results demonstrate its capability to reproduce cardiac contraction and demonstrate the feasibility of its use as an LV simulator rather than providing a comprehensive clinical study.

Active and passive beating ex-vivo heart platforms also offer realistic conditions to test medical devices [82,83]. However, the use of animal models and animal tissue in research raises ethical concerns [84], and there is a continuing debate about whether there will be a need for in-vivo experiments for implantable medical devices in the future [85]. The presented LV simulator has the potential to reduce the use of ex-vivo or acute animal tests for implantable devices, as the geometry of the LV on the system can be made anatomical, and it can replicate the torsion in the LV wall during contraction. Air pressure in the artificial muscles is regulated by a solenoid valve in the current design of the left ventricle simulator. An additional right ventricle in the system can be actuated by a separate solenoid valve synchronized with the solenoid valve driving the left ventricle. However, the air pressure in the artificial muscles or the design of the artificial muscles driving the right ventricle may be different from generating physiological right ventricular pressure and volume over a cardiac cycle. Developing a setup simulating synchronized left and right ventricles will be future work.

Conditions such as dilated or hypertrophic cardiomyopathies cause functional and anatomical changes in the LV. In dilated cardiomyopathy, the LV enlarges, and the LV muscle may be thinned, whereas hypertrophic cardiomyopathy may cause thickening in the LV muscle [86,87]. LV chambers can be made in different sizes and wall thicknesses to simulate dilated and hypertrophic cardiomyopathy, whereas pulse duplicators can only simulate LV function. LV rotation and twist increase with increasing age, whereas heart failure may also result in abnormal LV mechanics [88]. The effect of increasing age and contraction strength of the LV can be simulated by changing the angles and air pressure of the PAMs. Functional mitral regurgitation may be caused by dilation of the LV, resulting in loss of mitral leaflet coaptation [89]. Therefore, conditions associated with altered LV anatomy can also be evaluated in the developed LV simulator. Potential applications in this system also include testing of implants such as transcatheter mitral or aortic valve implants, especially where an anatomical shape is required for testing these devices [90].

Although pulse duplicators and mock circulatory systems have been widely used to test CF-LVAD support [91], data obtained from the pulse duplicators provide information about the hemodynamic signals in the cardiovascular system. A test setup with a realistic geometry of LV made of a flexible material would allow testing of further parameters, such as CF-LVAD cannula position [92]. Moreover, excessive pumping in CF-LVAD causes LV suction, which is an undesired pumping state with harmful effects [93]. The developed LV chamber will allow testing of clinical scenarios that cannot be tested in the pulse duplicators with pistons and rigid chambers. LV can also be built with an anatomical shape to simulate patient-specific conditions during CF-LVAD support. Moreover, devices used to cover the myocardium, such as the CorCap Cardiac Support Device, aim to correct ventricular remodeling [94] require anatomical models during the tests. The developed test setup has the potential to be used for such purposes.

Patient-specific geometries may be especially beneficial for simulating clinical conditions in pediatric patients. Diagnostic criteria for conditions such as hypertrophic cardiomyopathy are adapted from adults, as there is limited information available in the literature for children [95,96]. Therefore, testing clinical scenarios and developing implantable cardiac devices suitable for pediatric cohorts pose challenges [97]. The developed LV simulator has the potential to simulate pediatric hearts and may help to overcome the challenges mentioned. It should be noted that this study presents the design approach and the concept of the LV simulator.

Different scenarios, such as exercise, can be simulated by increasing the heart rate in the system. However, the LV model may need to be optimized to simulate those scenarios, because the mechanism for increased left ventricular filling and ejection during exercise is a result of increased contraction and relaxation velocity [98]. Moreover, the left ventricular mechanism of torsion and twist is also subject to debate [99]. For instance, LV systolic and diastolic twisting mechanics are reported to be impaired or improved during exercise [99]. Therefore, replicating left ventricular mechanics will be considered as future work.

There were the following limitations in this study. The stroke volume of the LV model was around 33 mL, whereas the residual volume was around 85 mL. Stroke volume usually ranges from 60 mL to 100 mL [78]. Therefore, the stroke volume remains below the physiological range for a healthy adult. However, end-diastolic volume in the developed LV simulator was around 118 mL, matching closely with adult end-diastolic left ventricular volume [78] for the given stroke and residual volumes. The maximal LV output was around 2.25 L/min because of low stroke volume in the LV simulator. Varying afterload and preload conditions were not tested in the experiments. A glycerin-water mixture with 40% glycerin and 60% water was used to match blood density and viscosity. Blood is a non-Newtonian fluid, whereas the circulation fluid in the LV simulator is typically a Newtonian fluid. The use of latex rubber for the LV wall and pneumatic artificial muscles will fail in long-term use due to fatigue. However, presented tests show that it is possible to complete tests such as hydrodynamic testing of mitral implants, as reported in [90]. Unlike electromechanical actuators, pneumatic systems may experience a latency between the control signal and the actual mechanical contraction [100]. Nevertheless, compliant actuation and flexible LV wall structure mimic the contraction of human myocardium. The repeatability of the hydrodynamic tests was not evaluated in the LV simulator. The experimental results presented in the manuscript are from one run for each setting.

## Conclusion

In this study, a one degree of freedom LV simulator system has been developed, and the hydrodynamic performance of the system has been tested. The LV simulator can generate average flow rates of up to 2.25 L/min, mean aortic pressures higher than 13 mmHg and systolic pressures higher than 30 mmHg. The twisting angle on the apex of the beating LV simulator was 21 degrees of rotation and 11 mm of apex shortening, similar to a native LV. The prototyped beating LV simulator is a promising preliminary platform and may contribute to the future development of physiologically relevant LV simulators with further refinement and validation. The wall and apex torsion motion of the prototyped beating LV simulator was parallel to the motion of the actual LV chamber.

## Supporting information

S1 File Video recording of the LV simulator. (ZIP)

S2 File Raw sensor data. (ZIP)

## References

[1] Jeong JH, Kim YM, Lee B, Hong J, Kim J, Woo SY. Design and evaluation of enhanced mock circulatory platform simulating cardiovascular physiology for medical palpation training. Appl Sci. 2020;10:5433. doi: 10.3390/app10165433

[2] Sedaghat A, Sinning J-M, Utzenrath M, Ghalati PF, Schmitz C, Werner N, et al. Hydrodynamic performance of the medtronic CoreValve and the edwards SAPIEN XT transcatheter heart valve in surgical bioprostheses: an in vitro valve-in-valve model. Ann Thorac Surg. 2016;101(1):118–24. doi: 10.1016/j.athoracsur.2015.06.047 26363653

[3] Joseph R, Wu C, Yoganathan A. Setting standards: revised ISO 5840 series clarifies testing, evaluation procedures for cardiac valves. Biomed Instrum Technol. 2020;54:441–3. doi: 10.2345/0899-8205-54.6.441 33339035

[4] Rocchi M, Ingram M, Claus P, D’hooge J, Meyns B, Fresiello L. Use of 3D anatomical models in mock circulatory loops for cardiac medical device testing. Artif Organs. 2023;47(2):260–72. doi: 10.1111/aor.14433 36370033

[5] Pulse duplicator system user manual. Vivitro Labs Inc.; 2011.

[6] Baloa LA, Boston JR, Antaki JF. Elastance-based control of a mock circulatory system. Ann Biomed Eng. 2001;29(3):244–51. doi: 10.1114/1.1355275 11310786

[7] Yu Y-C, Gopalakrishnan S. Elastance control of a mock circulatory system for ventricular assist device test. 2009 American Control Conference; 2009. p. 1009–14. 10.1109/acc.2009.5160629

[8] Schampaert S, Pennings KAMA, van de Molengraft MJG, Pijls NHJ, van de Vosse FN, Rutten MCM. A mock circulation model for cardiovascular device evaluation. Physiol Meas. 2014;35(4):687–702. doi: 10.1088/0967-3334/35/4/687 24622168

[9] Cappon F, Wu T, Papaioannou T, Du X, Hsu P-L, Khir AW. Mock circulatory loops used for testing cardiac assist devices: a review of computational and experimental models. Int J Artif Organs. 2021;44(11):793–806. doi: 10.1177/03913988211045405 34581613

[10] Imbrie-Moore AM, Zhu Y, Bandy-Vizcaino T, Park MH, Wilkerson RJ, Woo YJ. Ex vivo model of ischemic mitral regurgitation and analysis of adjunctive papillary muscle repair. Ann Biomed Eng. 2021;49(12):3412–24. doi: 10.1007/s10439-021-02879-9 34734363 PMC9466294

[11] Zhou W. In vitro simulation of mitral valve therapies [Ph.D.]. UCL (University College London); 2020. Available from: https://discovery.ucl.ac.uk/id/eprint/10098927/

[12] Ochsner G, Amacher R, Amstutz A, Plass A, Schmid Daners M, Tevaearai H, et al. A novel interface for hybrid mock circulations to evaluate ventricular assist devices. IEEE Trans Biomed Eng. 2013;60(2):507–16. doi: 10.1109/TBME.2012.2230000 23204266

[13] Bozkurt S, van Tuijl S, Schampaert S, van de Vosse FN, Rutten MCM. Arterial pulsatility improvement in a feedback-controlled continuous flow left ventricular assist device: an ex-vivo experimental study. Med Eng Phys. 2014;36(10):1288–95. doi: 10.1016/j.medengphy.2014.07.005 25066581

[14] Menne MF, Grossmann B, Schmitz-Rode T, Steinseifer U. Passive beating heart platform for testing, training and teaching of transcatheter therapies. Structural Heart. 2019;3:56. doi: 10.1080/24748706.2019.1588551

[15] de Hart J, de Weger A, van Tuijl S, Stijnen JMA, van den Broek CN, Rutten MCM, et al. An ex vivo platform to simulate cardiac physiology: a new dimension for therapy development and assessment. Int J Artif Organs. 2011;34(6):495–505. doi: 10.5301/IJAO.2011.8456 21725931

[16] Pelgrim GJ, Das M, Haberland U, Slump C, Handayani A, van Tuijl S, et al. Development of an ex vivo, beating heart model for CT myocardial perfusion. Biomed Res Int. 2015;2015:412716. doi: 10.1155/2015/412716 26185756 PMC4491382

[17] Leopaldi AM, Vismara R, van Tuijl S, Redaelli A, van de Vosse FN, Fiore GB, et al. A novel passive left heart platform for device testing and research. Med Eng Phys. 2015;37(4):361–6. doi: 10.1016/j.medengphy.2015.01.013 25666402

[18] Gorzalczany SB, Rodriguez Basso AG. Strategies to apply 3Rs in preclinical testing. Pharmacol Res Perspect. 2021;9:e00863. doi: 10.1002/prp2.863 PMC8491455 34609088

[19] Rocchi M, Papangelopoulou K, Ingram M, Bekhuis Y, Claessen G, Claus P, et al. A patient-specific echogenic soft robotic left ventricle embedded into a closed-loop cardiovascular simulator for advanced device testing. APL Bioeng. 2024;8(2):026114. doi: 10.1063/5.0203653 38812756 PMC11136518

[20] Davies J, Thai MT, Sharma B, Hoang TT, Nguyen CC, Phan PT, et al. Soft robotic artificial left ventricle simulator capable of reproducing myocardial biomechanics. Sci Robot. 2024;9(94):eado4553. doi: 10.1126/scirobotics.ado4553 39321276

[21] Davies J, Nicotra E, Zhu K, Nguyen CC, Sharma B, Ji A. A soft robotic model for simulating heart valve disease and cardiac interventions. Adv Sci. 2023;n/a:e16667. doi: 10.1002/advs.202516667 PMC13170261 41721571

[22] Gulbulak U, Ertas A. Finite element driven design domain identification of a beating left ventricular simulator. Bioengineering (Basel). 2019;6(3):83. doi: 10.3390/bioengineering6030083 31540196 PMC6784146

[23] Brouns R. Left ventricular wall motion abnormalities: What can they tell us about stroke recurrence? Neurology. 2017;88(6):510–1. doi: 10.1212/WNL.0000000000003591 28077496

[24] Triposkiadis F, Giamouzis G, Boudoulas KD, Karagiannis G, Skoularigis J, Boudoulas H, et al. Left ventricular geometry as a major determinant of left ventricular ejection fraction: physiological considerations and clinical implications. Eur J Heart Fail. 2018;20(3):436–44. doi: 10.1002/ejhf.1055 29105899

[25] Rupp LC, Bergquist JA, Zenger B, Gillette K, Narayan A, Tate JD, et al. The role of myocardial fiber direction in epicardial activation patterns via uncertainty quantification. Comput Cardiol (2010). 2021;48:10.23919/cinc53138.2021.9662950. doi: 10.23919/cinc53138.2021.9662950 35449765 PMC9020927

[26] Choi JY, Cha J, Jung JM, Seo WK, Oh K, Cho KH, et al. Left ventricular wall motion abnormalities are associated with stroke recurrence. Neurology. 2017;88:586–94. doi: 10.1212/WNL.0000000000003588 28077495

[27] Warfield JN. Binary matrices in system modeling. IEEE Trans Syst, Man, Cybern. 1973;SMC-3(5):441–9. doi: 10.1109/tsmc.1973.4309270

[28] Warfield JN. Developing subsystem matrices in structural modeling. IEEE Trans Syst, Man, Cybern. 1974;SMC-4(1):74–80. doi: 10.1109/tsmc.1974.5408523

[29] Ertas A, Rohman J, Chillakanti P, Baturalp T. Transdisciplinary collaboration as a vehicle for collective intelligence: a case study of engineering design education. Int J Eng Educ. 2015;31:1526–36.

[30] Sonnenblick EH. Force-velocity relations in mammalian heart muscle. Am J Physiol. 1962;202:931–9. doi: 10.1152/ajplegacy.1962.202.5.931 13915199

[31] Klabunde R. Cardiovascular physiology concepts. Lippincott Williams & Wilkins; 2011.

[32] Wang J, Khoury DS, Yue Y, Torre-Amione G, Nagueh SF. Left ventricular untwisting rate by speckle tracking echocardiography. Circulation. 2007;116(22):2580–6. doi: 10.1161/CIRCULATIONAHA.107.706770 17998458

[33] Geyer H, Caracciolo G, Abe H, Wilansky S, Carerj S, Gentile F, et al. Assessment of myocardial mechanics using speckle tracking echocardiography: fundamentals and clinical applications. J Am Soc Echocardiogr. 2010;23(4):351–69; quiz 453–5. doi: 10.1016/j.echo.2010.02.015 20362924

[34] Cheung Y. The role of 3D wall motion tracking in heart failure. Nat Rev Cardiol. 2012;9(11):644–57. doi: 10.1038/nrcardio.2012.128 22945330

[35] Evangelista A, Nardinocchi P, Puddu PE, Teresi L, Torromeo C, Varano V. Torsion of the human left ventricle: experimental analysis and computational modeling. Prog Biophys Mol Biol. 2011;107(1):112–21. doi: 10.1016/j.pbiomolbio.2011.07.008 21791224

[36] Shaw SM, Fox DJ, Williams SG. The development of left ventricular torsion and its clinical relevance. Int J Cardiol. 2008;130(3):319–25. doi: 10.1016/j.ijcard.2008.05.061 18678418

[37] Veress AI, Raymond GM, Gullberg GT, Bassingthwaighte JB. Left ventricular finite element model bounded by a systemic circulation model. J Biomech Eng. 2013;135(5):54502. doi: 10.1115/1.4023697 24231963 PMC3705858

[38] Hui L, Pemberton J, Hickey E, Li XK, Lysyansky P, Ashraf M, et al. The contribution of left ventricular muscle bands to left ventricular rotation: assessment by a 2-dimensional speckle tracking method. J Am Soc Echocardiogr. 2007;20(5):486–91. doi: 10.1016/j.echo.2006.10.012 17484988 PMC1978201

[39] Fan L, Yao J, Yang C, Tang D, Xu D. Infarcted left ventricles have stiffer material properties and lower stiffness variation: three-dimensional echo-based modeling to quantify in vivo ventricle material properties. J Biomech Eng. 2015;137(8):081005. doi: 10.1115/1.4030668 25994130 PMC4462863

[40] Rankin LS, Moos S, Grossman W. Alterations in preload and ejection phase indices of left ventricular performance,. Circulation. 1975;51(5):910–5. doi: 10.1161/01.cir.51.5.910 1122594

[41] Torrent-Guasp F, Kocica MJ, Corno AF, Komeda M, Carreras-Costa F, Flotats A, et al. Towards new understanding of the heart structure and function. Eur J Cardiothorac Surg. 2005;27(2):191–201. doi: 10.1016/j.ejcts.2004.11.026 15691670

[42] Kocica MJ, Corno AF, Carreras-Costa F, Ballester-Rodes M, Moghbel MC, Cueva CNC, et al. The helical ventricular myocardial band: global, three-dimensional, functional architecture of the ventricular myocardium. Eur J Cardio-Thorac Surg. 2006;29:S21–40. doi: 10.1016/j.ejcts.2006.03.011 16563790

[43] Van Der Smissen B, Claessens T, Verdonck P, Van Ransbeeck P, Segers P. Design of an artificial left ventricular muscle: an innovative way to actuate blood pumps? Artif Organs. 2009;33(6):464–8. doi: 10.1111/j.1525-1594.2009.00750.x 19473142

[44] Roche ET, Wohlfarth R, Overvelde JTB, Vasilyev NV, Pigula FA, Mooney DJ, et al. A bioinspired soft actuated material. Adv Mater. 2014;26(8):1200–6. doi: 10.1002/adma.201304018 24227698

[45] Hanson BM, Levesley MC, Watterson K, Walker PG. Hardware-in-the-loop-simulation of the cardiovascular system, with assist device testing application. Med Eng Phys. 2007;29(3):367–74. doi: 10.1016/j.medengphy.2006.05.010 16815728

[46] Alazmani A, Keeling D, Walker P, Abbas S, Jaber O, Watterson K. Introducing a hardware-in-the-loop simulation of the cardiovascular system. 2012:153–8. doi: 10.1109/BioRob.2012.6290799

[47] Bashir M, Rajendran P. A review on electroactive polymers development for aerospace applications. J Intell Mater Syst Struct. 2018;29:3681–95. doi: 10.1177/1045389X18798951

[48] Tozzi P, Michalis A, Hayoz D, Locca D, von Segesser LK. Artificial muscle for end-stage heart failure. ASAIO J Am Soc Artif Intern Organs. 2012;58(2):103–8. doi: 10.1097/MAT.0b013e3182452f00 22370680

[49] Harary F, Norman RZ, Cartwright D. Structural models: an introduction to the theory of directed graphs. Wiley; 1965.

[50] Duperrin J-C, Godet M. Méthode de hiérarchisation des éléments d’un système: essai de prospective du système de l’énergie nucléaire dans son contexte sociétal. report, Centre national de l’entrepreneuriat(CNE); CEA; 1973. 63 p., figures. Available from: https://hal-lara.archives-ouvertes.fr/hal-02185432

[51] Mandal A, Deshmukh SG. Vendor selection using interpretive structural modelling (ISM). Int J Oper Prod Manag. 1994;14:52–9. doi: 10.1108/01443579410062086

[52] Veronesi F, Corsi C, Caiani EG, Sarti A, Lamberti C. Tracking of left ventricular long axis from real-time three-dimensional echocardiography using optical flow techniques. IEEE Trans Inf Technol Biomed. 2006;10(1):174–81. doi: 10.1109/titb.2005.855535 16445262

[53] El Missiri AM, El Meniawy KAL, Sakr SAS, Mohamed ASE. Normal reference values of echocardiographic measurements in young Egyptian adults. Egypt Heart J. 2016;68(4):209–15. doi: 10.1016/j.ehj.2016.01.002

[54] Bozkurt S. Mathematical modeling of cardiac function to evaluate clinical cases in adults and children. PLoS One. 2019;14(10):e0224663. doi: 10.1371/journal.pone.0224663 31671136 PMC6822734

[55] Baturalp TB, Bozkurt S. Design and analysis of a polymeric left ventricular simulator via computational modelling. Biomimetics. 2024;9. doi: 10.3390/biomimetics9050269 PMC11117906 38786479

[56] Brindise MC, Busse MM, Vlachos PP. Density and viscosity matched newtonian and non-newtonian blood-analog solutions with PDMS refractive index. Exp Fluids. 2018;59(11):173. doi: 10.1007/s00348-018-2629-6 31745378 PMC6863344

[57] Kim S, Prasad B, Kim JK. Alignment of microbeads using spinning helical minichannel cartridge. J Korean Soc Vis. 2016;14: 38–45. doi: 10.5407/JKSV.2016.14.3.038

[58] Bombardini T, Gemignani V, Bianchini E, Venneri L, Petersen C, Pasanisi E, et al. Diastolic time - frequency relation in the stress echo lab: filling timing and flow at different heart rates. Cardiovasc Ultrasound. 2008;6:15. doi: 10.1186/1476-7120-6-15 18426559 PMC2365937

[59] Baturalp TB, Ertas A. State of the art mock circulation loop and a proposed novel design; 2015. Available from: https://www.semanticscholar.org/paper/State-of-the-Art-Mock-Circulation-Loop-and-a-Novel-Baturalp-Ertas/f0a63ecfd68ad13f13fd7a7a9a346c0b478f0eb9

[60] Attri R, Dev N, Sharma V. Interpretive structural modelling (ISM) approach: an overview. 2013;2:3–8.

[61] Zipes DP, Libby P, Bonow RO, Mann DL, Tomaselli GF. Braunwald’s heart disease: a textbook of cardiovascular medicine, 2-volume Set. 11th ed. Philadelphia (PA): Elsevier; 2018.

[62] Forman EH, Gass SI. The analytic hierarchy process—an exposition. Oper Res. 2001;49:469–86. doi: 10.1287/opre.49.4.469.11231

[63] Antony J. Design of experiments for engineers and scientists. Amsterdam; Boston: Elsevier; 2014.

[64] Mitchell M. An introduction to genetic algorithms. Cambridge (MA): MIT Press; 2001.

[65] Song Z, Borazjani I. The role of shape and heart rate on the performance of the left ventricle. J Biomech Eng. 2015;137(11):114501. doi: 10.1115/1.4031468 26312776

[66] Ingels NB Jr. Myocardial fiber architecture and left ventricular function. Technol Health Care. 1997;5(1–2):45–52. doi: 10.3233/thc-1997-51-205 9134618

[67] Buckberg G, Mahajan A, Saleh S, Hoffman JIE, Coghlan C. Structure and function relationships of the helical ventricular myocardial band. J Thorac Cardiovasc Surg. 2008;136(3):578–89, 589.e1-11. doi: 10.1016/j.jtcvs.2007.10.088 18805255

[68] Buckberg G, Hoffman JIE, Mahajan A, Saleh S, Coghlan C. Cardiac mechanics revisited: the relationship of cardiac architecture to ventricular function. Circulation. 2008;118(24):2571–87. doi: 10.1161/CIRCULATIONAHA.107.754424 19064692

[69] Trumble DR, McGregor WE, Kerckhoffs RCP, Waldman LK. Cardiac assist with a twist: apical torsion as a means to improve failing heart function. J Biomech Eng. 2011;133(10):101003. doi: 10.1115/1.4005169 22070328

[70] Sengupta PP, Tajik AJ, Chandrasekaran K, Khandheria BK. Twist mechanics of the left ventricle: principles and application. JACC Cardiovasc Imaging. 2008;1(3):366–76. doi: 10.1016/j.jcmg.2008.02.006 19356451

[71] Omar AMS, Vallabhajosyula S, Sengupta PP. Left ventricular twist and torsion. Circ Cardiovasc Imaging. 2015;8(6):e003029. doi: 10.1161/circimaging.115.003029 25991575

[72] Kormányos Á, Kalapos A, Domsik P, Lengyel C, Forster T, Nemes A. Normal values of left ventricular rotational parameters in healthy adults-Insights from the three-dimensional speckle tracking echocardiographic MAGYAR-Healthy Study. Echocardiography. 2019;36(4):714–21. doi: 10.1111/echo.14285 30801756

[73] Tavakoli V, Sahba N. Assessment of age-related changes in left ventricular twist by 3-dimensional speckle-tracking echocardiography. J Ultrasound Med. 2013;32:1435–41. doi: 10.7863/ultra.32.8.1435 23887954

[74] Sun JP, Lam Y-Y, Wu C-Q, Yang XS, Guo R, Kwong JSW, et al. Effect of age and gender on left ventricular rotation and twist in a large group of normal adults--a multicenter study. Int J Cardiol. 2013;167(5):2215–21. doi: 10.1016/j.ijcard.2012.06.017 22727965

[75] Grüner Sveälv B, Fritzon G, Andersson B. Gender and age related differences in left ventricular function and geometry with focus on the long axis. Eur J Echocardiogr. 2006;7(4):298–307. doi: 10.1016/j.euje.2005.06.008 16039910

[76] Roche ET, Horvath MA, Wamala I, Alazmani A, Song S-E, Whyte W, et al. Soft robotic sleeve supports heart function. Sci Transl Med. 2017;9(373):eaaf3925. doi: 10.1126/scitranslmed.aaf3925 28100834

[77] Lorenzon L, Beccali G, Cianchetti M. A preliminary study on an innovative soft robotic artificial heart ventricle. 2023 IEEE International Conference on Soft Robotics (RoboSoft). 2023. p. 1–8. 10.1109/RoboSoft55895.2023.10121955

[78] Hall JE, Hall ME. Guyton and hall textbook of medical physiology. Philadelphia (PA): Elsevier; 2021.

[79] Sack KL, Dabiri Y, Franz T, Solomon SD, Burkhoff D, Guccione JM. Investigating the role of interventricular interdependence in development of right heart dysfunction during LVAD support: a patient-specific methods-based approach. Front Physiol. 2018. Available from: https://www.frontiersin.org/articles/10.3389/fphys.2018.00520 10.3389/fphys.2018.00520 PMC5962934 29867563

[80] Thygesen K, Alpert JS, White HD. Universal definition of myocardial infarction. Circulation. 2007;116:2634–53. doi: 10.1161/CIRCULATIONAHA.107.187397 17951284

[81] Murphy SP, Ibrahim NE, Januzzi JL Jr. Heart failure with reduced ejection fraction: a review. JAMA. 2020;324(5):488–504. doi: 10.1001/jama.2020.10262 32749493

[82] Bozkurt S, van Tuijl S, van de Vosse FN, Rutten MCM. Arterial pulsatility under phasic left ventricular assist device support. Biomed Mater Eng. 2016;27(5):451–60. doi: 10.3233/BME-161599 27885993

[83] Granegger M, Mahr S, Horvat J, Aigner P, Roehrich M, Stoiber M, et al. Investigation of hemodynamics in the assisted isolated porcine heart. Int J Artif Organs. 2013;36(12):878–86. doi: 10.5301/ijao.5000257 24362896

[84] Doke SK, Dhawale SC. Alternatives to animal testing: a review. Saudi Pharm J. 2015;23(3):223–9. doi: 10.1016/j.jsps.2013.11.002 26106269 PMC4475840

[85] Sorguven E, Bozkurt S, Baldock C. Computer simulations can replace in-vivo experiments for implantable medical devices. Phys Eng Sci Med. 2021;44(1):1–5. doi: 10.1007/s13246-021-00978-4 33559037

[86] Mahmaljy H, Yelamanchili VS, Singhal M. Dilated cardiomyopathy. In: StatPearls. Treasure Island (FL): StatPearls Publishing; 2023. Available from: http://www.ncbi.nlm.nih.gov/books/NBK441911/28722940

[87] Basit H, Brito D, Sharma S. Hypertrophic cardiomyopathy. In: StatPearls. Treasure Island (FL): StatPearls Publishing; 2023. Available from: http://www.ncbi.nlm.nih.gov/books/NBK430788/

[88] Phan TT, Shivu GN, Abozguia K, Gnanadevan M, Ahmed I, Frenneaux M. Left ventricular torsion and strain patterns in heart failure with normal ejection fraction are similar to age-related changes. Eur J Echocardiogr. 2009;10(6):793–800. doi: 10.1093/ejechocard/jep072 19502618

[89] Vajapey R, Kwon D. Guide to functional mitral regurgitation: a contemporary review. Cardiovasc Diagn Ther. 2021;11(3):781–92. doi: 10.21037/cdt-20-277 34295705 PMC8261742

[90] Bozkurt S, Preston-Maher GL, Torii R, Burriesci G. Design, analysis and testing of a novel mitral valve for transcatheter implantation. Ann Biomed Eng. 2017;45(8):1852–64. doi: 10.1007/s10439-017-1828-2 28374279 PMC5527080

[91] Bozkurt S, van de Vosse FN, Rutten MCM. Enhancement of arterial pressure pulsatility by controlling continuous-flow left ventricular assist device flow rate in mock circulatory system. J Med Biol Eng. 2016;36:308–15. doi: 10.1007/s40846-016-0140-1 27441034 PMC4935750

[92] Imamura T, Narang N, Nitta D, Fujino T, Nguyen A, Kim G, et al. Optimal cannula positioning of HeartMate 3 left ventricular assist device. Artif Organs. 2020;44(12):e509–19. doi: 10.1111/aor.13755 32557769

[93] Rabi SA, D’Alessandro DA. Suction event after LVAD placement. In: Sundt TM, Cameron DE, Lee ME, editors. Near misses in cardiac surgery. Cham: Springer International Publishing; 2022. p. 179–81. doi: 10.1007/978-3-030-92750-9_45

[94] Mann DL, Kubo SH, Sabbah HN, Starling RC, Jessup M, Oh JK, et al. Beneficial effects of the CorCap cardiac support device: five-year results from the Acorn Trial. J Thorac Cardiovasc Surg. 2012;143(5):1036–42. doi: 10.1016/j.jtcvs.2011.06.014 21762937 PMC3790142

[95] Lipshultz SE, Law YM, Asante-Korang A, Austin ED, Dipchand AI, Everitt MD, et al. Cardiomyopathy in children: classification and diagnosis: a scientific statement from the American Heart Association. Circulation. 2019;140(1):e9–68. doi: 10.1161/CIR.0000000000000682 31132865

[96] Bakaya K, Paracha W, Schievano S, Bozkurt S. Assessment of cardiac dimensions in children diagnosed with hypertrophic cardiomyopathy. Echocardiography. 2022;39(9):1233–9. doi: 10.1111/echo.15437 35978451

[97] Burki S, Adachi I. Pediatric ventricular assist devices: current challenges and future prospects. Vasc Health Risk Manag. 2017;13:177–85. doi: 10.2147/VHRM.S82379 28546755 PMC5437969

[98] Stoylen A, Wisløff U, Slørdahl S. Left ventricular mechanics during exercise: a Doppler and tissue Doppler study. Eur J Echocardiogr. 2003;4(4):286–91. doi: 10.1016/s1525-2167(03)00008-8 14611824

[99] Drury CT, Bredin SS, Phillips AA, Warburton DE. Left ventricular twisting mechanics and exercise in healthy individuals: a systematic review. Open Access J Sports Med. 2012;3:89–106. doi: 10.2147/OAJSM.S32851 24198592 PMC3781904

[100] Dihovicni DN. Distributed and time-delay analyse of pneumatic signals in long pipelines. In: Fernández-Izquierdo MÁ, Muñoz-Torres MJ, León R, editors. Modeling and simulation in engineering, economics, and management. Berlin, Heidelberg: Springer; 2013. p. 250–6. doi: 10.1007/978-3-642-38279-6_27

